# Red Light and 5% Aminolaevulinic Acid (5%) Inhibit Proliferation and Migration of Dysplastic Oral Keratinocytes via ROS Production: An In Vitro Study

**DOI:** 10.3390/gels9080604

**Published:** 2023-07-26

**Authors:** Tania Vanessa Pierfelice, Milos Lazarevic, Dijana Mitic, Nadja Nikolic, Milena Radunovic, Giovanna Iezzi, Adriano Piattelli, Jelena Milasin

**Affiliations:** 1 Department of Medical, Oral and Biotechnological Sciences, University G. d’Annunzio of Chieti-Pescara, 66100 Chieti, Italy; tania.pierfelice@unich.it (T.V.P.); gio.iezzi@unich.it (G.I.); 2 Department of Microbiology and Immunology, School of Dental Medicine, University of Belgrade, Dr Subotica 8, 11000 Belgrade, Serbia; milena.radunovic@stomf.bg.ac.rs; 3 Department of Human Genetics, School of Dental Medicine, University of Belgrade, Dr Subotica 8, 11000 Belgrade, Serbia; milos.lazarevic@stomf.bg.ac.rs (M.L.); dijana.trisic@stomf.bg.ac.rs (D.M.); nadja.nikolic@stomf.bg.ac.rs (N.N.); jelena.milasin@stomf.bg.ac.rs (J.M.); 4 School of Dentistry, Saint Camillus International University of Health and Medical Sciences, 00131 Rome, Italy; 5 Facultad de Medicina, UCAM Universidad Catolica San Antonio de Murcia, 30107 Guadalupe, Spain

**Keywords:** 5-ALA, LED, photodynamic therapy, dysplastic oral keratinocytes (DOK), ROS, necrosis

## Abstract

Undiagnosed and untreated oral precancerous lesions often progress into malignancies. Photodynamic therapy (PDT) might be a minimally invasive alternative to conventional treatments. 5-aminolevulinic acid (5-ALA) is one of the most commonly used photosensitizers in PDT, and it is effective on many cancer types. However, its hydrophilic characteristic limits cell membrane crossing. In the present study, the effect of a newly formulated gel containing 5% 5-ALA in combination with red light (ALAD-PDT) on a premalignant oral mucosa cell line was investigated. The dysplastic oral keratinocyte (DOK) cells were incubated with ALAD at different concentrations (0.1, 0.5, 1, and 2 mM) at two different times, 45 min or 4 h, and then irradiated for 7 min with a 630 nm LED (25 J/cm^2^). MTT assay, flow cytometry, wound healing assay, and quantitative PCR (qPCR) were performed. ALAD-PDT exerted inhibitory effects on the proliferation and migration of DOK cells by inducing ROS and necrosis. mRNA analysis showed modulation of apoptosis-related genes’ expression (TP53, Bcl-2, survivin, caspase-3, and caspase-9). Furthermore, there was no difference between the shorter and longer incubation times. In conclusion, the inhibitory effect of the ALAD-PDT protocol observed in this study suggests that ALAD-PDT could be a promising novel treatment for oral precancerous lesions.

## 1. Introduction

Oral cancer is the sixth most common cancer, with a high worldwide incidence [1]. Oral precancerous lesions involve the epithelium of the mouth and may be at risk in developing oral cancer, which are associated with a high mortality rate [2]. Therefore, early detection and early treatment of precancerous lesions are fundamental for increasing patient survival and, consequently, their quality of life. Photodynamic therapy (PDT) may be a valid alternative to conventional treatment methods such as surgery, radiotherapy, and chemotherapy [3]. Compared to these cancer treatment options, PDT has the advantage of being minimally invasive; it does not induce drug resistance or have systemic side effects like chemotherapy [4]. PDT uses a light-sensitive compound, a photosensitizer, which selectively accumulates inside cells such as bacteria or cancer cells [5,6]. The photochemical reaction occurs when the photosensitizer is excited by visible light at specific wavelengths and reacts with molecular oxygen. This photodynamic reaction leads to singlet oxygen and free radical production, which damages the target cells [5]. One of the most commonly used drugs in PDT clinical practice is 5-aminolevulinic acid (5-ALA), a component of the heme biosynthesis chain, acting as a precursor of the endogenous photosensitizer protoporphyrin IX (PpIX) [7]. Although 5-ALA is effective in many cancer types, its hydrophilic characteristic limits its ability to cross the cell membrane [8]. This limiting property has driven the formulation of novel chemicals to better convey the active ingredient to cells. ALADENT, a gel containing 5% of 5-ALA, is a lipophilic compound owing to its formulation based on a poloxamer mixture [9]. Hence, ALADENT can enhance the intracellular accumulation of the photosensitizer, protoporphyrin IX (PpIX), into target cells because its formulation is more lipophilic than 5-ALA itself. ALADENT, combined with a red light at 630 nm (ALAD-PDT), has shown efficacy against oral microorganisms in in vitro and in vivo studies [10,11,12,13]. Notably, the ALAD-PDT protocol has also demonstrated antibacterial effects against oral biofilm [11,14,15]. ALAD-PDT was originally designed as a strategy for oral microorganism infection control in the context of periodontal disease. Its efficacy in treating oral precancerous lesions is unexplored. Considering that an inhibitory effect of 5-ALA on the viability of premalignant cells was reported [16,17,18], the aim of this study was to examine the effect of ALAD-PDT on the proliferation and migration of the premalignant oral mucosa cell line DOK, along with potential mechanisms involved in it.

## 2. Results and Discussion

### 2.1. ALAD-PDT Treatment Induced Cell Death in DOK

**Figure 1:**
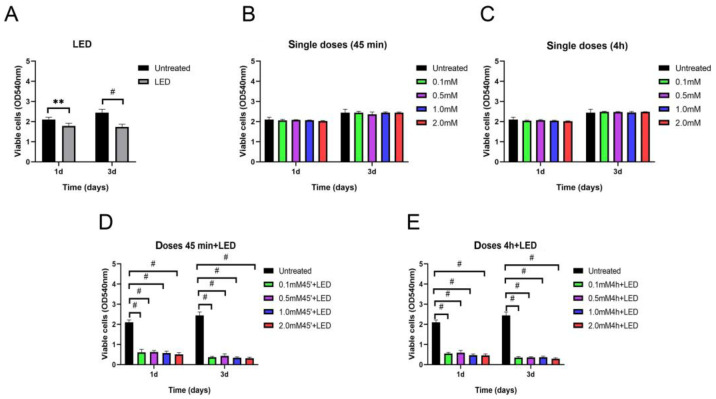
Analysis of cell viability in DOK cells, measured with the MTT assay after 1 day and 3 days the photodynamic protocol (ALAD-PDT). (**A**) Cells irradiated with LED alone compared to untreated cells; (**B**) cells incubated for 45 min with different concentrations (mM) of ALADENT; (**C**) cells incubated for 4 h with different concentrations (mM) of ALADENT; (**D**) cells incubated for 45 min with different concentrations (mM) of ALADENT and then irradiated with LED for 7 min; (**E**) cells incubated for 4 h with different concentrations (mM) of ALADENT and then irradiated with LED for 7 min. Data are presented as mean ± SD; *n* = 6. ** *p* < 0.01, # *p* < 0.0001.

**Figure 2:**
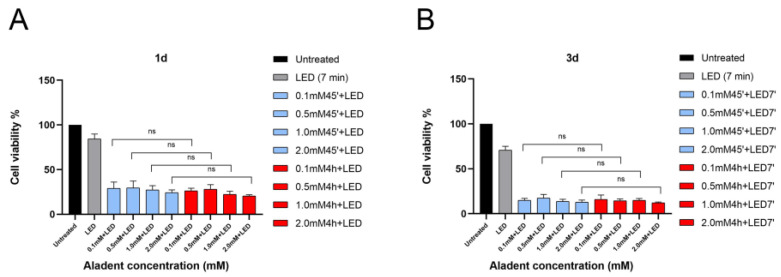
Analysis of cell viability in DOK cells expressed in percentage. The 45 min groups were individually compared with 4 h groups. Data are presented as mean ± SD; *n* = 6. ns = 45 min group (light blue bars vs. 4 h group (red bars) indicates non-significative result. (**A**) 1d and (**B**) 3d.

The cytotoxic activity of ALAD-PDT on oral precancerous DOK cells was assessed at 24 h and 72 h after the treatment. Given that it was previously demonstrated that the survival of oral cancer cells could be inhibited by 5-ALA in a dose-dependent manner, in this study, ALAD was applied at different concentrations (0.1, 0.5, 1, and 2 mM/mL) with and without irradiation (Figure 1). One of the strengths of the ALAD-PDT protocol is the relatively short time necessary for the entire procedure. Namely, it takes 45–60 min for gel application and 7 min for irradiation with a LED device. However, previous studies have shown the antitumoral effects of 5-ALA incubated for 4 h because of the slow permeability of the 5-aminolevulinic acid into the target cells [16,17,18]. In particular, ALADENT is a sol–gel, liquid at room temperature (sol) and semi-solid at body temperature (gel). It was formulated to improve muco-adhesion properties and to speed up the penetration of 5-ALA across the cellular membrane [9]. The class of carriers, particularly convenient for topical applications, that can confer higher permeability to the acid (5-ALA) refer to a triblock copolymer made of polyethylene oxide (PEO) and polypropylene oxide (PPO) known as poloxamers. Collaud et al. showed how a thermosetting formulation based on this poloxamers mixture was more suitable than creams in releasing 5-ALA ester derivates [19]. This poloxamers mixture showed an excellent compatibility with other chemicals and high solubilization capacity for different drugs [19,20]. The effect of the ALADENT protocol at different concentrations on normal cells, such as human fibroblasts and human osteoblasts, was already demonstrated in our previous studies [21,21]. Therefore, we compared the sensitivity of DOK cells incubated with ALAD for 45 min and DOK cells incubated with ALAD for 4 h (Figure 2). At day 1, compared to untreated cells, ALAD without irradiation did not cause changes in cell viability, whereas irradiation alone induced a certain cytotoxic effect, and the viability of DOK cells was reduced to 84.69 ± 3.77%. However, the optimum inhibition of cell viability was observed when cells were subjected to the whole (ALAD-PDT) treatment (Figure 1A–E and Figure 2A). A reduction in cell viability was observed in a dose-dependent manner. The following percentages of viable cells were recorded: 29.09 ± 3.61%, 29.55 ± 3.06%, 27.14 ± 3.19%, and 24.08 ± 2.91% after a 45 min incubation of DOK with the gel at concentrations of 0.1 mM, 0.5 mM, 1 mM, and 2 mM of ALAD, respectively, and exposure to light (Figure 1D and Figure 2A). The viability of cells further decreased for all concentrations (0.1 mM, 0.5 mM, 1 mM, and 2 mM) when the incubation time was prolonged (4 h), being 26.19 ± 2.73%, 28.03 ± 3.32%, 22.24 ± 2.67%, and 21.85 ± 2.64%, respectively (Figure 1E and Figure 2A). No statistically significant differences in viability reduction could be observed between cells incubated for 45 min and for 4 h (Figure 2A). Further inhibition of cell viability was observed at 3 days post-treatment (Figure 2B). Applying LED alone resulted in 70.84 ± 3.22% of viable cells, while the greatest inhibition efficiencies were achieved by combining ALAD gel with LED (Figure 1A). At all doses (0.1, 0.5, 1, and 2 mM), the viability of DOK cells decreased below 20% after ALAD-PDT, regardless of the incubation time. The values for viable cells treated for 45 min were 14.93 ± 2.03%, 17.68 ± 2.93%, 13.81 ± 2.41%, and 12.95 ± 2.45%, while the viability of cells after 4 h of incubation was 14.41 ± 2.51%, 14.81 ± 2.54%, 14.97 ± 2.69%, and 12.04 ± 2.44%, respectively (Figure 2B). Thus, the viability assay results showed that ALAD does not exhibit cytotoxic effects on DOK cells without light. Other studies using 5-ALA have also reported this finding in oral precancerous, oral squamous carcinoma, breast cancer, and hepatocellular carcinoma cells [9,16,19]. On the other hand, light alone inhibited the viability of cells; the reduction in viable cells after 7 min of irradiation was approximately 30% at 3 days post-treatment (Figure 2B). This is in line with an in vitro study that described the anti-proliferation properties of LED at 630 nm on human synoviocytes irradiated for 15 min [20]. Recently, another study described the inhibitory activities of a 660 nm LED in various breast cancer-origin cell lines irradiated for 30 min [21]. The present study showed that ALAD-PDT had a remarkable dose- and time-dependent inhibitory effect on the proliferation of DOK cells, with the optimum effect achieved with the highest dose of ALAD-PDT (2 mM). Similar anti-proliferative activity of 5-ALA on oral precancerous cell lines has already been reported. However, because of its poor membrane permeability, the incubation time of cells with 5-ALA in the previous studies was 4 h or more [16,17,18,22,23]. ALAD gel was formulated precisely to increase the penetrability of 5-ALA into tissues and cells. The results of DOK cells incubated for 45 min with ALAD and the results of DOK cells incubated for a longer time (4 h) were compared (Figure 2A,B). As hypothesized, no statistically significant differences could be revealed between the two incubation times, even though cells incubated with ALAD for 4 h showed a greater decrease in viability.

### 2.2. ALAD-PDT Treatment Inhibited Migration Capacity of DOK

**Figure 3:**
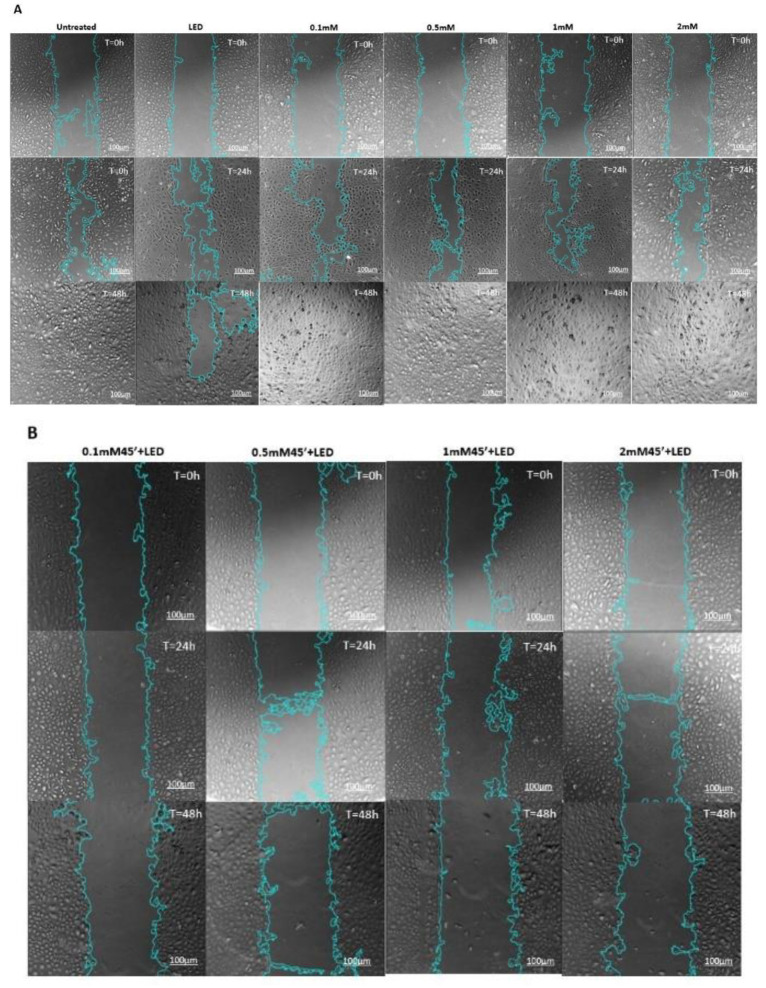

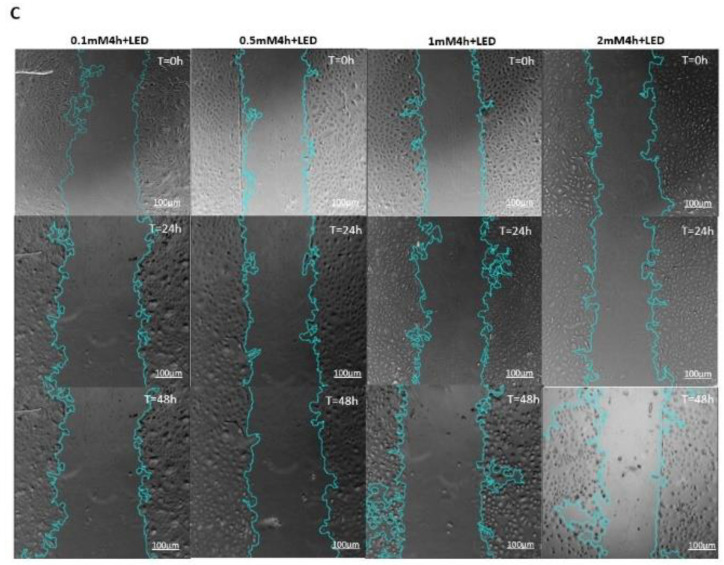
Images from wound healing assays using phase contrast microscopy at different time points (0 h, 24 h, and 48 h). Scale bar = 100 µm. Representative images of scratched and recovering wounded areas (marked by blue lines) on confluence monolayers of DOK cells were analyzed using the ImageJ software. (**A**) Untreated cells and any treatments alone. (**B**) The migration rate of cells treated with ALAD-PDT for 45 min. (**C**) Cell migration rate after the whole treatment for 4 h.

**Figure 4:**
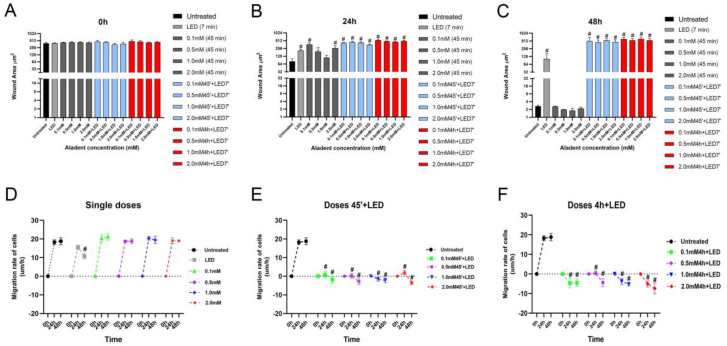
The wound healing assay revealed differences in the migration rates of DOK cells. (**A**–**C**) wound area μm^2^ at different time points (0 h, 24 h, and 48 h). (**D**–**F**) Summary graph showing migration rates of DOK cells in the presence of different treatments. Data are presented as mean ± SD; *n* = 3. ** *p* < 0.01 vs. untreated; # *p* < 0.0001 vs. untreated.

A wound healing assay was performed to verify whether the photodynamic treatment could decrease the metastatic potential of the tested precancerous cell lines, as shown in Figure 3. None of the doses of ALAD exhibited any inhibitory effect on the migration rate of DOK cells, and during the experiment, the cell-free area progressively diminished. In the cells treated with ALAD without light, wound closure began after 24 h, and complete closure occurred after 48 h of cultivation, as was observed in the control cells. LED alone was able to partially inhibit the closure of the wound (Figure 3A). Previous studies have shown the opposite effects of irradiation with LED or laser on promoting or inhibiting cellular migration, depending on the experimental settings, i.e., dosimetric parameters such as wavelength, energy, and irradiation frequency [24,25]. Based on the dose–response relationship between phototreatment and cancer cells, Schalch et al. found that oral squamous cell carcinoma irradiated with a 660 nm laser (40 mW; 4 J/cm^2^) demonstrated greater wound closure capacity than cells irradiated with a 780 nm laser (70 mW; 4 J/cm^2^) [24,26]. On the contrary, Henriques et al. observed a greater invasion potential of oral squamous cell carcinoma cells irradiated with a 660 nm laser using two energy densities (0.5 and 1.0 J/cm^2^) compared to the control group [27]. In our study, DOK cells’ migration rate was noticeably decreased after ALAD-PDT. ALAD-PDT significantly enhanced the width of the wound by enhancing the cell-free area (Figure 4). At 48 h post-treatment, the scratches appeared larger than the initial wounds (Figure 3B,C). Beyond the wound area, a reduction in attached cells was observed after 48 h of treatment with the highest dose of ALAD (2 mM), incubated for 4 h (Figure 3C). As illustrated in the graphs, the quantification of wound area (μm^2^) in time confirmed the ALAD-PDT protocol’s inhibitory effect (Figure 4A–C). The rate of cells moving towards the scratched area revealed that untreated cells and cells treated with ALAD alone migrated faster to close the gap of a scratch than LED-treated cells (Figure 4D). On the contrary, ALAD-PDT significantly reduced cell migration of DOK cells compared with untreated cells (Figure 4E,F). The greatest reduction in cell migration was observed in DOK cells incubated for 4 h with all doses of ALADENT plus light (Figure 4F).

### 2.3. ALAD-PDT Treatment Induced ROS Production in DOK

**Figure 5:**
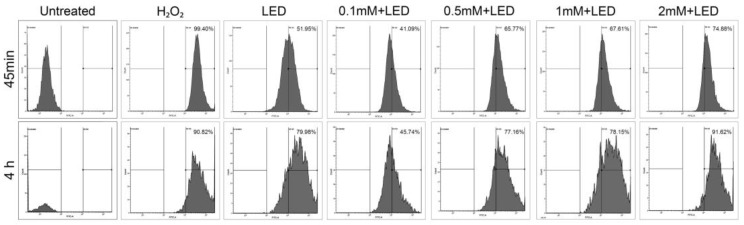
Changes in intracellular ROS after the ALAD-PDT protocol. The detection of ROS levels in DOK cells exposed to different doses of ALAD-PDT and LED alone was determined by flow cytometry. The percentage of H_2_O_2_ in each group was reported. Untreated cells were considered negative controls, and H_2_O_2_-treated cells were considered positive controls.

Based on the mechanism of PDT, the death of cells is due to oxidative damage, i.e., the increment of ROS production [5]. Flow cytometry experiments with DCFH2-DA fluorescein were used to measure cellular ROS formation and investigate whether differences in intracellular ROS levels appeared in DOK cells treated with ALAD-PDT at different concentrations and incubation times. Although ROS predominantly include superoxide anion (O_2_^−^), hydrogen peroxide (H_2_O_2_), and hydroxyl radical (OH) [28], H_2_O_2_ represents the most stable, being able to cross the cell membrane through passive and diffusion mechanisms, and it plays a crucial role in ROS-dependent signaling [29]. Thus, in this study, H_2_O_2_-treated cells were considered positive controls, and only the percentage of H_2_O_2_ as reference ROS was reported in the flow cytometry analysis. As shown in Figure 5, the analysis revealed that ALAD-PDT generated ROS in a photosensitizer dose-dependent manner. It should be mentioned that the same trend was observed for both incubation times. In cells incubated with ALAD for 45 min, the generated ROS levels were lower than those generated in H_2_O_2_-treated cells. Similarly, the percentage of ROS produced in cells incubated with ALAD for 4 h was lower than in H_2_O_2_-treated cells, except for 2 mM+LED. This result perfectly reflects the trend of viability results, indicating that the amount of ROS generated can impact the effects of PDT. The cellular ROS formation under LED light was measured because LED light also provoked cytotoxic effects on DOK cells. Interestingly, the obtained ROS values were lower than in H_2_O_2_-treated cells but higher than in 0.1 mM+LED-treated cells. This could be due to cellular ROS formation by LED light, not only from the mitochondria but also via NADPH oxidase-dependent mechanisms [26]. Considering that oxidative stress is lethal when the activity of the oxidants prevails over the antioxidants, it could be interesting to investigate in a future study the antioxidant agents in DOK cells.

### 2.4. ALAD-PDT Treatment Mainly Induced Necrosis in DOK

**Figure 6:**
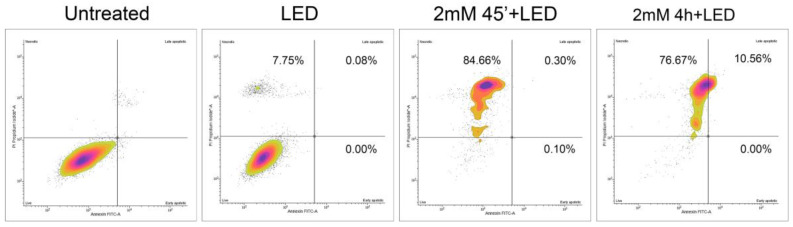
Annexin V-PI dual-staining assay evaluated apoptosis/necrosis in DOK cells induced by different treatments. The plots were divided into four regions, and the percentage of apoptotic/necrotic was reported.

It is well known that PDT can lead to apoptosis, necrosis, and autophagy [30]. Double staining with Annexin V/PI was performed 24 h after treatment to assess the influence of ALAD-PDT on the death of DOK cells. Compared to the control group, a necrosis-inducing effect was observed in cells treated with 2 mM-ALAD combined with LED. As shown in Figure 6, the proportions of necrotic DOK cells were 84.66% and 76.67% after 45 min and 4 h, respectively. A small proportion of apoptotic cells (10.56%) was observed after 4 h of incubation with ALAD-PDT. LED irradiation alone induced a small number of necrotic cells compared to the control group. The results suggest that necrosis is the main type of cell death induced by ALAD-PDT. Even though necrosis is less observed than apoptosis in PDT studies, it was reported that the cell type, the subcellular localization of the photosensitizer, the type and site of generated ROS, and the light dose applied to activate the photosensitizer are factors and parameters determining which will be the fate of the cell, necrosis or apoptosis, following PDT [30,31,32]. A recent study reported that 5-ALA-based photodynamic therapy can induce necrosis and apoptosis in pathologic proliferative skin cells [33]. Another study demonstrated singlet oxygen production after 5-ALA-PDT-induced RIP3-dependent necrotic pathway activation [34].

### 2.5. ALAD-PDT Treatment Induced Changes in Apoptosis-Related Genes

**Figure 7:**
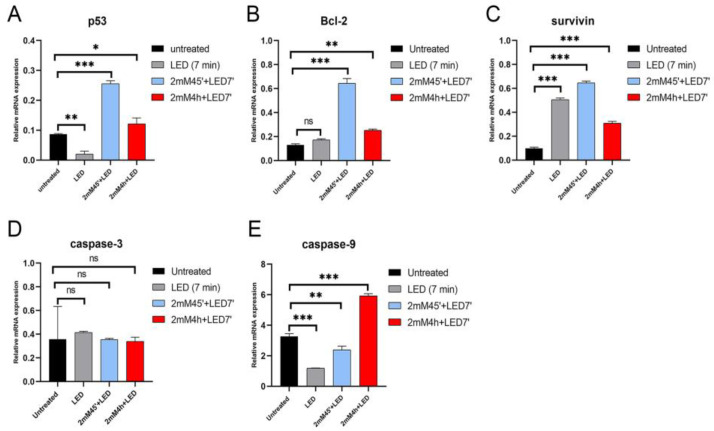
RT-PCR analysis evaluated the effect of ALAD-PDT treatment on (**A**) p53, (**B**) Bcl-2, (**C**) Survivin, (**D**) caspase-3, and (**E**) caspase-9, apoptotic-related markers in DOK cells. Data are presented as mean ± SD; *n* = 3. * *p* < 0.05 vs. untreated; ** *p* < 0.01 vs. untreated; *** *p* < 0.001 vs. untreated; ns—non significant.

To further investigate the mechanism behind the ALAD-PDT-induced cell death, the expression of apoptosis-related genes such as TP53, caspase-3, caspase-9, Bcl-2, and Survivin was studied, and the results were reported in the Figure 7. TP53, Bcl-2, and Survivin were significantly upregulated in ALAD-PDT-treated DOK cells, with the highest values in cells incubated for 45 min (Figure 7A–C). No significant modulation in the expression of caspase-3 could be observed in ALAD-PDT-treated cells compared to control (Figure 7D). In contrast, the expression of caspase-9 showed an opposite trend depending on the incubation time. Indeed, the level of caspase-9 mRNA was downregulated in ALAD-PDT-treated cells incubated for 45 min but upregulated in ALAD-PDT-treated cells incubated for 4 h (Figure 7E). Each of these genes’ role in promoting apoptotic pathways is well documented [35]. However, recent studies suggest necrosis shares signaling pathways and genes with programmed cell death, apoptosis [36,37]. The tumor suppressor protein p53 is a stress-responsive transcription factor involved in biological functions such as cell cycle arrest, apoptosis, senescence, metabolic dynamics, and autophagy. Still, studies also revealed a role for p53 in the regulation of necrotic cell death by altering mitochondrial permeability [38,39], and it is known that the necrosis mechanism implies the formation of the mitochondrial permeability transition pore (MPTP) [40]. In a murine model, it was demonstrated that oxidative stress stimulates the translocation of p53 to the mitochondria to promote MPTP and, consequently, necrosis via the cyclophilin-D-p53 interaction [39]. This result was supported by different in vitro studies demonstrating CypD-p53 complex formation in MPTP-induced necrosis in neuronal, osteoblastic, and pancreatic cells [41,42,43]. There is evidence that overexpression of the anti-apoptotic Bcl-2 gene is a form of resistance to chemotherapeutic drugs in various cancer types [44]. However, a dual pattern of Bcl-2 overexpression was observed in PDT-treated cancer cells. For instance, a protective role against PDT-induced apoptosis [45] and an increased death rate in Bcl2-overexpressing head and neck cancer cells were reported [46,47]. Survivin also acts as an anti-apoptotic gene and, like Bcl-2, is a form of cancer cell resistance to chemotherapeutic drugs. Its suppressive ability is achieved via inhibition of caspase activity [48]. Studies showed that knockdown of the Survivin gene is sufficient to improve photokilling efficiency [49,50]. Caspases play an important role in the proteolysis-mediated activation of apoptotic proteins. The cleavage of nuclear proteins represents the events that characterize apoptosis; caspase-9 is considered the initiator, and caspase-3 is the effector [47]. Studies investigating PDT as an anticancer strategy reported both apoptosis and necrosis after PDT treatment via modulation of p53, Bcl-2, and caspase genes [31,46,51,52]. Meng P. et al. showed the ability of photodynamic treatment to inhibit oral squamous cell carcinoma viability by upregulating p53 [53]. In a recent study, necrosis was the main form of cell death in Foscan-photosensitized HT29 cells that overexpressed Bcl-2. In contrast, apoptosis was the main type of cell death in cells with upregulated caspase-3 [52]. Kessel D. reported the inhibition of procaspase-9 and procaspase-3 and the overexpression of Bcl-2 in porphyrin-induced necrotic cells. Kessel D. indicated the inactivation of procaspase-9 and procaspase-3 as the signal shifting in favor of necrosis [36]. This could explain the apoptotic cell population detected only in ALAD-PDT-treated cells incubated for 4 h, where caspase-9 was upregulated compared to ALAD-PDT-treated cells incubated for 45 min.

Altogether, the present study indicates that the photodynamic protocol ALAD-PDT can substantially decrease cell viability and cell migration of dysplastic oral keratinocytes via ROS production. Interestingly, necrosis appeared to be the dominant cell death after ALAD-PDT. The type and site of ROS generated in PDT-exposed cancer cells seem to be the decisive factors in promoting necrosis or apoptosis [36,37,54]. Gene expression analysis suggested that anti-apoptotic molecules generally suppress apoptotic pathways in ALAD-PDT-treated cells. This could be seen as a form of triggered resistance in DOK cells, but the ALAD-PDT protocol robustly photokilled cells via ROS- mediated necrosis.

## 3. Conclusions

In conclusion, the data showed the synergistic effect of combined ALADENT and LED in inhibiting cell proliferation and migration. This points to the ALAD-PDT protocol as a potential alternative treatment for oral precancerous lesions. The treatment can be repeated many times in the same area without systemic toxicity. However, only several aspects of the procedure were elucidated. Therefore, further studies are required to identify the molecular mediators involved in the cell death mechanisms in ALAD-PDT-exposed cancer cells.

## 4. Materials and Methods

### 4.1. Cell Culture

Dysplastic oral keratinocytes (DOK) (European Collection of Authenticated Cell Cultures, 94122104) were cultured in complete medium Dulbecco’s Modified Eagle Medium (DMEM) supplemented with 10% fetal bovine serum, 100 U/mL streptomycin-penicillin solution (all from Thermo Fisher Scientific, Waltham, MA, USA). Cell culture medium was supplemented with 0.5 µg/mL hydrocortisone (Thermo Fisher Scientific, Waltham, MA, USA) as previously reported [55]. It was reported in different studies that hydrocortisone, as a supplement in cell culture media, can enhance the proliferation of various cell lines including keratinocytes, epithelial, endothelial, lung embryonic fibroblasts, and (HEL) cells. It was demonstrated that hydrocortisone can also promote the initial attachment of cells on the substratum surface of cells [56,57]. A recent study evaluated the effect of hydrocortisone in 2D and 3D cell culture of human laryngeal carcinoma cells (HEp-2). In this study, the exposure of hydrocortisone promoted the spreading of cells on substrate and increased cell proliferation, but the effect occurred within 24 h, indicating that the influence of supplement is for a short period just for the initial phase of cell culture [58]. Another recent in vitro study demonstrated that the addition of hydrocortisone in human laryngeal carcinoma (HEp-2) cell culture is able to influence chemotherapeutic treatment but cannot influence photodynamic treatment [59]. Cells were grown in a humidified atmosphere under standard conditions (37 °C and 5% CO_2_).

### 4.2. Aladent Treatment Protocol

DOK cells were incubated for 45 min (45 min) or for 4 h (4 h) with different concentrations (mM/mL) of the gel containing 5% of 5-aminolevilinic acid (ALAD), commercialized as ALADENT by ALPHA Strumenti s.r.l. (Melzo, MI, Italy) and then irradiated for 7 min (7 min) with an LED device TL-01 (ALPHA Strumenti s.r.l., Melzo, MI, Italy). Different concentrations (0.1, 0.5, 1.0, 2.0 mM/mL) of ALADENT gel were obtained by diluting the gel in serum-free DMEM medium. The LED device emits a red light at 630 nm with a power density of 380 mW/cm^2^, with a light energy density of 25 J/cm^2^ [10]. The diameter of the light spot was 1 cm in diameter, and it was used at a working distance of 0.5 cm. All irradiation procedures have been performed in the dark condition under a laminar flow hood. Protective glasses and gloves were worn for exposure to red light.

### 4.3. MTT Assay

Cells (5 × 10^3^ per well) were seeded in 96 wells/plate and grown for 24 h. Cells were then incubated with different concentrations of ALAD gel (0.1 mM/mL, 0.5 mM/mL, 1.0 mM/mL, and 2.0 mM/mL) in serum-free medium and then irradiated as described in the ALADENT treatment protocol. In addition, groups of cells treated only with different concentrations of ALAD alone (not subjected to light irradiation) were included. Untreated cells were considered the positive control group; cells treated with light alone for 7 min were assigned to the LED group. After 24 h and 72 h treatment, the medium was removed, and 100 μL of 5 mg/mL MTT solution (Sigma-Aldrich, Taufkirchen, Germany) was added and incubated for 4 h. After removing the supernatant, the formazan crystals were dissolved in 100 μL of dimethyl sulfoxide (Sigma-Aldrich) by shaking for 15 min at 37 °C. Utilizing a microplate reader (RT-2100c, Rayto, Shenzhen, China), optical density (OD) was measured at 540 nm. The cell viability (%) was calculated using the formula of Equation (1) as previously reported [60]:

ODsample/ODblank × 100(1)

### 4.4. Wound Healing Assay

Cells were seeded in a 24-well plate at a concentration of 1 × 10^5^ cells/well and left until they reached 80% confluence. A scratch was conducted with a 200 μL sterile pipette tip. Detached cells were washed away with PBS (1×). Cells were then treated as protocol. At each time point (0 h, 24 h, 48 h), the plate was placed under a phase-contrast microscope to observe the migration rate. The selected regions of interest were observed using an inverted microscope on a 10× objective (Primovert Zeiss, Berlin, Germany). To analyze the images using the Wound Healing Size Tool, an ImageJ software 1.48 version (NIH, Bethesda, MD, USA) plugin was used. It allows the quantification of wound area (μm^2^) in images obtained from a wound healing assay. The rate of cell migration (RM) was calculated according to Equation 2 as previously described [61]:

RM = (W_0_ − W_t_)/t (2)

where W_0_ is the average of the initial wound width, W_t_ is the average of the final wound width both in μm, and t is the time span of the assay in hours.

### 4.5. Reactive Oxygen Species (ROS) Detection

DOK cells were divided into the LED irradiation alone group, ALAD at each concentration (0.1 mM/mL, 0.5 mM/mL, 1.0 mM/mL, and 2.0 mM/mL) incubated for 45 min or 4 h combined with LED irradiation. For this experiment, untreated cells were considered negative control, and cells stimulated with H_2_O_2_ to a final concentration of 100 μM were considered positive control. Cells (1 × 10^5^) were treated and then stained in the dark with 20 μM 2,7-Dichlorofluorescin diacetate (DCF-DA) (Sigma-Aldrich, St. Louis, MO, USA) for 30 min at 37 °C. The level of intracellular ROS was determined by flow cytometry (BD Biosciences, Franklin Lakes, NJ, USA). The mean fluorescence intensity was determined using BD FACS/MELODY software https://www.bdbiosciences.com/en-eu/products/instruments/flow-cytometers/research-cell-sorters/bd-facsmelody accessed on 17 April 2023.

### 4.6. Annexin V/PI Staining

Dok cells were divided into the LED irradiation alone group, ALAD-PDT incubated for 45 min or 4 h, and the control group. Given that the highest concentration of ALAD resulted in the optimum concentration from the MTT assay, 2 mM/mL was chosen to perform Annexin V/PI staining. DOK were cultured into 24-well plates (1 × 10^5^ per well). After treatment, Annexin staining for detecting apoptosis was performed with an Annexin V–FITC Kit (Invitrogen, Thermo Fisher Scientific) according to the manufacturer’s instructions. Propidium iodide (PI) was used to detect necrotic or late apoptotic cells. Staining was analyzed by flow cytometry (BD Biosciences), and the results were presented in a two-dimensional dot plot of PI versus Annexin V–FITC. The plots were divided into four regions corresponding to: (a) viable cells, negative for both probes (PI/FITC −/−; Q3); (b) apoptotic cells, PI-negative and Annexin-positive (PI/FITC −/+; Q1); (c) late apoptotic cells, PI- and Annexin-positive (PI/FITC +/+; Q2); (d) necrotic cells, PI-positive and Annexin-negative (PI/FITC +/−; Q4).

### 4.7. RNA Isolation and Reverse Transcription

Total RNA was extracted from cells using the TRIzol reagent (Invitrogen, Thermo Fisher Scientific). The total RNA concentration was measured using a microvolume spectrophotometer (BioSpec–nano Microvolume UV–Vis Spectrophotometer; Shimadzu Scientific Instruments, Columbia, MD, USA). An oligo d(T) primer and TRANSCRIPTME Reverse Transcriptase (Blirt, S.A., Gdańsk–Poland) were used to synthesize cDNA from 4 µg of total RNA [62].

### 4.8. Gene Expression Analysis of Apoptotic Markers

Dok cells were divided into the LED alone group, the ALAD-PDT group incubated for 45 min or 4 h, and the control group. Additionally, for Annexin V/PI analysis, the highest concentration of ALAD (2 mM) was chosen to perform gene expression analysis. Real-time quantitative polymerase chain reaction (qPCR) was performed using the first strand cDNA, 0.2 μM forward and reverse primers, and a SensiFAST SYBR Hi–ROX Kit (Bioline, London, UK). The expression of the following markers was analyzed: p53, caspase-3, caspase-9 (pro-apoptotic), Bcl-2, and Survivin (anti-apoptotic). The housekeeping gene, glyceraldehyde-3-phosphate dehydrogenase—GAPDH, was used as a reference. Relative gene expression values were calculated using the 2^−ΔCt^ method [63]. The sequences of all primers used in this study are given in Table 1.

### 4.9. Statistical Analysis

GraphPad Prism ver. 8.0 (GraphPad Software, Inc., La Jolla, CA, USA) was used for all statistical analyses. Quantitative data were presented as the mean ± SD from three independent experiments. Comparisons among the groups were assessed using one-way analysis of variance (ANOVA) with post hoc Tukey’s test for multiple comparisons. Two-tailed *p* < 0.05 was considered statistically significant. Data from flow cytometry analysis were reported as percentages (%).

## 5. Patents

Aladent gel is protected by a patent (PCT/IB2018/060368, 12.19.2018).

## Figures and Tables

**Table 1:** List of primers used in gene expression analysis.

Primer Name	Sequence (F) *	Sequence (R) *
TP53	ATGTTTTGCCAACTGGCCAAG	TGAGCAGCGCTCATGGTG
Bcl-2	ATGTGTGTGGAGAGCGTCAACC	TGAGCAGAGTCTTCAGAGACAGCC
Caspase-3	TGTTTGTGTGCTTCTGAGCC	CACGCCATGTCATCATCAAC
Caspase-9	CATTTCATGGTGGAGGTGAAG	GGGAACTGCAGGTGGCTG
Survivin	TCTGGCGTAAGATGATGG	GAAATAAGTGGGTCTGAAGTG
GAPDH	ATGGGGAAGGTGAAGGTCG	GGGGTCATTGATGGCAACAATA

* Primer sequences are indicated as forward (F) and reverse (R).

## Data Availability

Not applicable.
